# Impact of Contact With Nature on the Wellbeing and Nature Connectedness Indicators After a Desertic Outdoor Experience on Isla Del Tiburon

**DOI:** 10.3389/fpsyg.2022.864836

**Published:** 2022-06-03

**Authors:** Glenda Garza-Terán, Cesar Tapia-Fonllem, Blanca Fraijo-Sing, Daniela Borbón-Mendívil, Lucía Poggio

**Affiliations:** ^1^Programs of Master and Doctorate in Psychology, University of Sonora, Hermosillo, Mexico; ^2^Departamento de Psicología Social, del Trabajo y Diferencial, Universidad Complutense de Madrid, Madrid, Spain

**Keywords:** nature connectedness, wellbeing, natural environment, contact with nature, desertic environments

## Abstract

Nature connectedness is determined by the representation individuals have about themselves within nature. This concept is often studied in relation to the direct contact individuals have with natural environment, which according to some studies have demonstrated to generate positive effects by fostering a feeling of connecting and bonding with nature, as well as improving their wellbeing. The main focus of this study was to calculate and assess the relation between Nature Connectedness and wellbeing of participants. The methodological approach of this research reaches quantitative data comparing results obtained from both samples, as well as correlations between the variables. The sample for this study was composed by two groups of university students (*M* = 25 years old). Both contrast group (*n* = 32) and experience group (*n* = 29) filled the questionnaire in two separate moments and in different environments. First data collection moment for both groups was held inside a university classroom. A second moment of data collection was carried out after a month from the first application, having the contrast group answer the questionnaire on a classroom again whilst the experience group responded it during an excursion to Isla Del Tiburon in Northwestern Mexico after performing some recreational activities being totally immersed in a local desertic environment. Questionnaire was composed by a 6 point Likert type scale measuring Nature Connectedness through concepts such as Nature relatedness and Love and care for the natural, as well as Subjective and Psychological Wellbeing of participants. Results show that both wellbeing and Nature Connectedness are positively influenced by performing activities out in the natural environment. This work was also conducted in response to the need to understand the full extent of Contact and Connectedness to nature, carrying out an exploratory study in desertic settings when much of the early work centers around the study of these variables in green nature environments.

## Introduction

Individuals often receive positive effects after being in contact with nature. Experimental studies have shown how this contact arise while interacting with plants, animals, natural views or walking on nature (Morita et al., 2007; Hinds and Sparks, 2008; Müller et al., 2009; Duerden and Witt, 2010; Collado and Corraliza, 2016; Izenstark et al., 2021). This contact brings direct effects on each individual affective, emotional, and psychological strands, among others.

Certainly, contact with nature occurs while an individual interacts with any natural component or is surrounded by a natural environment. Certainly, these interactions are diverse, and they may include various activities such as mountain biking on the forest, indoor hiking on a virtual simulated jungle or even working on an office with a panoramic view of the greenery outside. Consequently, humans benefit from this interaction enhancing their connectedness to nature, integrity, vitality, wellbeing amongst others. Thus, contact with nature is considered as a key predictor to an individual level of Nature Connectedness (NC) (Mayer and Frantz, 2004; Olivos et al., 2011).

Contact with nature has been frequently studied in contexts such as psychology and environmental education, these seek to explain the attainment of benefits of an affective connection to the natural environment (Millar and Millar, 1996; Hinds and Sparks, 2008; Müller et al., 2009; Duerden and Witt, 2010; Collado and Corraliza, 2016).

There exists a very extensive literature on the topic of the relationship between environmental identity and self-identity. A large body of work has approached this variables with different measures, concluding that there is indeed a strong correlation between the aforementioned elements and furthermore, with them also being related to different measures of nature connectedness (Brügger et al., 2011; Tam, 2013; Restall and Conrad, 2015; Martin and Czellar, 2016; Olivos and Clayton, 2017; Balundė et al., 2019).

Additionally, widely reported studies can be found about the beneficial or detrimental effects of the human-nature relationship regarding the degree of connection and lifestyle choices of individuals (Seymour, 2016). According to Kaplan and Kaplan (1989) the fondness people may display for natural environments vary according to individual differences. Said preferences range from birdwatching and contact with animals and plants to walking or “Forest bathing” (Translated from *shinrin- yoku*, Kotera et al., 2020).

This connection is referred as a significant predictor of intentions that conduce people to interact with the natural environment in a certain way. Moreover, NC can have a varying influence on the development of the environmental concern, as is required to perform a comparison between having a direct experience with nature and another with an indirect contact (Collado and Corraliza, 2016). Olivos and Aragonés (2014), to study NC of individuals and Inclusion of Nature in Self (INS) both in natural and built environments, established that there is a distinct augmentation in people NC after being exposed on a natural environment.

Previous studies assessing Contact with nature or natural environment attitudes, usually take place on green backgrounds (e.g., parks, woods, forest). However, this study is executed on the Isla Del Tiburon, a climate with characteristics of the arid Sonoran Desert located in northwestern Mexico (see Supplementary Photographs and Map).

This study aims to explore individuals’ nature connectedness that are not in contact with nature and therefore, do not have any kind of interaction outdoors at the moment of being assessed, compared to those who are in direct contact with nature and are evaluated while being exposed to outdoor activity considering that the environment presents extreme conditions (e.g., high exposure to sun, dry weather, hot temperatures, lack of sanitary amenities, pathway trails on the island).

The present study has the purpose of analyzing the level of nature connectedness and wellbeing individuals display when surrounded by this particular scenery. Our work seeks to test whether contrast and experience groups present significant differences regarding their levels of nature connectedness and Psychological Wellbeing. We have hypothesized that outdoor activity and being in direct contact with nature on Isla Del Tiburon can positively enhance individual nature connectedness. Accordingly entailing, the experience group will develop a higher sense of feeling closer to nature, a better perception of feeling satisfied with life, as well as having positive emotions.

### Contact With Nature: An Approach to Nature Connectedness

Being connected to nature or the NC construct is the sense and level of belonging humans have with the natural world (Schultz et al., 2004). This term is the result of analyzing the self-nature bond, and it suggests a perception perceived as an extension of the cognitive representation that all humans have, given an individual belief of being part of nature. This determines the concern Individuals with higher sense of kindness will develop and the circumstances under which they will come to action toward nature. This conceptualization gained attention in the 1950s, when there was a surge in the interest in meditation and the natural environment, its concern, and the bonding people have with it; but it was not until the 1990s when scientists coined the term.

This concept of NC is enriched by several proposals that comprise the concept such as Nature Relatedness (NR), which Nisbet et al. (2009) suggested a new construct based on the relatedness or relationship with nature to describe the levels of connectedness individuals have with natural world. This proposition constitutes the appreciation and comprehension people have toward other living things on the planet and surpasses the principles of environmentalism as it is a more dynamic concept that does not simply suggest pleasure or love for nature, but a complex understanding of the importance of the natural environment in diverse levels and aspects. The concept of NR describes individual levels of NC and is similar to the fundamental concept of ecology, which consists of a notion of self-construction included in nature.

Another idea that integrates to NC is Love and Care for Nature (LCN) by Perkins (2010), which estimates the level of feeling connected with nature and the personal fulfillment attained by it. This arises after analyzing an affective aspect about the relationship between nature and human beings contributing to “environmental altruism,” and is built upon developing an integral love and care for nature, as well as recognizing its intrinsic value and acquiring a sense to protect it.

According to Schultz et al. (2004), the NC that emanates after bonding the self and nature is related to the way individuals see themselves within nature, in addition to a set of motivational believes that will promote the emergence of environmental behaviors. This means that each person will estimate nature from their own cognitive representation followed by acquiring a type of environmental concern, being subsequently motivated to act in a certain way toward nature (Olivos and Aragonés, 2014). This idea is known as Inclusion of Nature in Self (INS) by Schultz (2001).

### Nature Connectedness

Several types of environmental concerns and situations have been addressed as individual motivators of nature’s behavior caused by an internal belief in how people position themselves within the natural world (Schultz et al., 2004). On one side, there is the individual that accepts itself as an element apart from nature and considers people to be exempt of the nature kingdom by considering themselves as superior to plants and animals. Oppositely, there is the individual that perceives itself to be a part of nature equal to animals, and that the same rights that humans have, apply just as well as they do to other living organisms. NC represents the integrity of an individual with the natural world, following Leopoldo’s idea that people need to feel part of a natural environment if they desire to properly engage in environmental problems or feel related to nature.

By comprehending the NC concept as an identification of how individuals perceive themselves toward the natural environment, authors suggest this condition is also important when estimating the human-nature relationship. Natural environments are often related on significance to contact with nature, which genuinely portrays a crucial aspect on the individual level of connectedness to nature (Nisbet et al., 2009). Regarding these environmental interventions, researchers indicate substantial contact with nature might enhance self- perception within nature, being connected to a natural world and as a part of it from an early age (Barrable and Booth, 2020; Pirchio et al., 2021).

### Nature Connectedness and Wellbeing

The benefits of being exposed to natural environments seem to be mediated by the sense of belonging, integrity, and NC (Mayer and Frantz, 2004; Olivos et al., 2011). Environmental Psychology has studied the effects of NC in relation to variables such as wellbeing, restoration, stress, or fatigue. The study of the psycho-emotional and physical benefits that stem from this contact has led to relevant findings like Kasap et al. (2021) who assert that nature as a whole has enormous effects on the human being cognitive functioning as improvements on wellness, stress, and anxiety (Fong et al., 2018; Ameli et al., 2021; Reese et al., 2021). Izenstark et al. (2021) also reference this by associating being in a natural environment to emotional wellness benefits, whether it be adults or children (Bowler et al., 2010; McMahan and Estes, 2015; Ward et al., 2016). PW is also significantly influenced by visiting urban parks and green areas or performing activities in them. These spaces include reserves, fields, communal gardens, and natural conservation areas (Roy et al., 2012; Loureiro and Veloso, 2014). Conversely, Song et al. (2017) performed a study (*n* = 20) where they implemented forest bathes in which participants increased their levels of calmness and relaxation after the activity while Pirchio et al. (2021) performed a study (*n* = 407) here they ascertained participants’ NC rising after completing a program of outdoor activities in a natural area.

### Wellbeing and Natural Environments

When studying the effects of NC related to variables such as wellbeing, the focus is to investigate if the environment on which interactions occur brings some effects (e.g., happiness, vitality, relaxation) and if so, in which way. For this, two philosophies try to measure and conceptualize the term by promoting a debate with theoretical and practical implications.

While Ryan and Deci (2001) define wellbeing as the optimal functioning and psychological experience of individuals, Subjective wellbeing (SW) studies the reason and way people positively experience their lives while including cognitive judgments such as affective reactions (Diener, 1994). De Sade believed that this kind of search for a sense and pleasure is the goal of human life (Ryan and Deci, 2001). Contrastingly, Psychological wellbeing (PW) has its philosophical origins in the works of Aristotle, who characterized eudaimonia because of people’s lives according to their own values and their self-realization (Waterman, 2008; Pritchard et al., 2020). Thus, it is important to mention that individuals that perceive themselves as being more intricately connected to nature often register higher indices of eudaimonic wellness, particularly regarding their personal growth. Studies acknowledge different results after measuring wellbeing and NC because of differences in aspects within wellbeing that are considered when relating it to NC (Howell and Passmore, 2013; Capaldi et al., 2014; Pritchard et al., 2020); such is the case of results obtained after studying eudaimonic wellbeing with NC, since they confirm the bond between these two aspects may be stronger than the one between NC and hedonic wellbeing (Howell et al., 2011; Capaldi et al., 2014).

Exposure to nature may have a positive influence on psychological constructs as well, such as boredom, sympathy, wellbeing, and liveliness (Morita et al., 2007; Lim et al., 2020) in addition to raising personal levels of energy and vitality (Ryan et al., 2010). Similarly, there is an improvement on expressing feelings or emotions while being surrounded by nature as proven by Kaplan and Talbot (1983), who claim that when people find themselves in wild environments they report feeling “alive” and engaged with nature, in conjunction with scoring notably higher relaxation indicators (Hansen et al., 2017; Lim et al., 2020).

Studies confirm that being outdoors and in contact with the natural environment is a way for people to satisfy their needs of having direct experiences in a natural world; while cognitive and emotional experiences are associated to having positive effects on their wellbeing (Tauber, 2012). In Singapore, Lim et al. (2020) implemented what is known as a “forest bathing” to measure exposure to the woods for a period and its effects. On this study (*n* = 51) participants gathered on a guided walk through the forest answering an instrument that included scales such as Nature Connectedness Scale (NCS), where the results were positive on people’ NC, as well as to some positive aspects in their emotions through PANAS (Positive and Negative Affect Schedule). Regarding subjective wellbeing scales, PANAS is used to determine people’s SW and often applied in experimental studies to evaluate changes in affections before and after some outdoor activity reporting benefits in affective aspects (Bowler et al., 2010; McMahan and Estes, 2015; Izenstark et al., 2021). Another common way to capture SW it is measuring through Satisfaction with life (National Research Council, 2013). Being exposed to nature or being outdoors a strong predictor from this concept (Kaplan, 1993; Biedenweg et al., 2017). The presence of natural elements (e.g., animals, plants, views) often create a greater life satisfaction on people at diverse environments; Kaplan (1993) found that satisfaction increased with a natural view from peoples’ home (Russell et al., 2013).

### Natural Environment: Tiburon Island

Simmons (1993) defines natural environment as everything that surrounds us that is not human; and from this definition spreads the dichotomy of what is a result of human influence and what remains untouched. Kaplan and Kaplan (1989) define the term “natural environment” and refer to it as “nature” encasing the meaning of a group of living and non-living elements that constitute a habitat composed by spaces and resources where there are no human being traces found. This idea corresponds to a continuum where one edge is the natural environment and the opposite is a built environment, according to Aragonés and Amérigo (2000).

In this same notion, Isla Del Tiburon is a natural environment, and it is located in the Gulf of California, in northwestern Mexico. It has a surface area of 1,208 km^2^ and is characterized by having a dry climate and two mountain ranges: Sierra Menor and Sierra Kun Kaak. Local vegetation is comprised over 298 species from the Sonoran Desert (Rojas et al., 2002). Moreover, it is a site of high biological productivity, with areas for nesting, and mating and breeding of marine species. It is the largest island in the country and has been labeled as “biosphere reserve.” In addition, it is part of a UNESCO World Heritage Site and belongs to one of the ethnic groups in the region called “Seri” (Konkaak), comprised by 700 people (II Conteo de Población y Vivienda in Acosta, 2002). This ethnic group bases its cultural manifestations tributing nature, sea, animals, and the different stages of human beings and life cycle. The island is part of Seri territory and is considered one of the last territories of the Sonoran Desert that remains untamed. Therefore, Isla Del Tiburon represents a perfect setting to assess what this study aims, considering closeness to the city (approximate a 50 miles drive) and being a desert virgin territory full of natural panoramas.

## Methodology

This study is performed under a quantitative methodological approach as it sustains a set of processes that implement techniques of quantitative measurement and statistical analysis. Furthermore, it is a quasi-experiment that focuses on the dependent variables that have been collected in pre-organized conditions in order to describe the way or reason of the presented situation or phenomena (Tamayo, 1980). This is a descriptive and comparative study analyzing situational variables, which consists of observing certain characteristics participants present in two different environments and two different periods of time: classroom and/or a natural environment (Island), 1 month after answering the initial questionnaire.

### Participants

Sample is composed by two student groups, both enrolled in the Psychology School Program at the University of Sonora in Mexico. The “Contrast group” corresponds to 32 students from 18 to 36 years old (*M* = 24 years old) enrolled in the undergraduate psychology program that were part of a course and were already gathered at the classroom while “Experience group” to 29 students aged 18 to 36 years old (*M* = 23 years old) composed by undergraduate and graduate students from the same program and were invited to the excursion. Both samples were openly invited to participate in this study and accepted voluntarily to be part of it.

### Instrument

A 68-item instrument was used and adapted on this study and is comprised by seven scales. Participants’ Subjective Wellbeing (SW) is studied with the Positive and Negative Affect Schedule (PANAS); it is a list of adjectives that name emotions as positive and negative affects of people in particular situations. It was designed by Watson et al. (1988) and consists of a total 20 items in Likert-type scale divided on two scales: PA and NA, which correspond to positive affects and the negative. This scale presented high internal consistency (α = 0.80). Correlation between the two affect factors (positive and negative) is low, ranging from -0.12 to -0.23, so they are interpreted as independent (Watson et al., 1988). This two-factor model demonstrated a good fit (Zevon and Tellegen, 1982; Crocker, 1997; Terracciano et al., 2003). PANAS Currently has been validated in Mexico (Robles and Páez, 2003 as shown in Moral, 2011).

Participants must choose from a range (1 = nothing–5 = very) if they feel inspired, anguished. Satisfaction with life Scale (SWLS) (Diener et al., 1985) was also used to measure SW, it consists of five items that evaluate the self-perception people have about how satisfied they are with their own lives. It contains statements such as “In most aspects, my life is how I want it to be” with a range of answers (1 = strongly disagree–7 = strongly agree).

To measure the NC variable the Nature Connectedness Scale (NCS) by Mayer and Frantz (2004) was used on its seven-item adaptation (Pasca et al., 2017), which measures the subjective cognitive connection between individuals and nature. NCS has accounted high internal consistency (α = 0.84) It contains statements such as “I often feel related with animals and plants with a range of answers” (1 = strongly disagree–7 = strongly agree).

Moreover, Love and Care for Nature Scale (LCN, α = 0.90) by Perkins (2010) was employed to report statements such as “I feel a deep love for nature” with a Likert-type 15 item scale (1 = strongly disagree to 7 = strongly agree).

Nature Relatedness Scale (NRS) created and validated by Nisbet et al. (2009) was also added as a Likert-type scale with 6 items (α = 0.87). This scale relates people and their preferences of subjects about nature and their level of comfort in the environment with statements such as “My connection to nature and the environment is a part of my spirituality” and “I always think about how my actions affect the environment” with a range of options (1 = strongly disagree–7 = strongly agree) to mark as their personal alignment.

Schultz’s Inclusion of Nature in Self (INS) (2001) one-item was added to measure construction of self within nature. INS consists of a range of options of seven pairs of circles, with left circle labeled as “Self” and the right one under “Nature.” Each group gradually overlap until being completely one circle option (1 = Self and Nature are totally separate–7 = Self and Nature represent a whole same circle). With this item, participants are asked to choose the pair of circles that best represents their sense of being connected to the natural world. This item could not receive a factor analysis as it is a single item instrument.

Wellbeing is measured with the Psychological Wellbeing Scale (PWS) by Ryff (1989) where 29 items relate to people life achievements as “I feel good when I think about what I have done in the past and what I expect to do in the future” and a range of answers (1 = strongly disagree–7 = strongly agree).

Additionally, sociodemographic items were added (e.g., gender, age).

### Procedure

Two different groups conformed this research, one contrast and one experience. The experience group was previously selected after they signed in over an open-call that took place in the Psychology department at the Universidad de Sonora where the public was invited to an experience of contact with nature. Contrast group was comprised by undergraduate students from the Psychology department as well as those who chose not to partake in the outdoor activities.

Data collection process was executed in two different settings. First, Contrast and Experience groups responded to a printed instrument at a university classroom while sitting at their desks on regular schedule taking 15 min to answer it. At that moment, participants had not had any contact with nature at all while answering the test. Second data collection moment was held a month after the contrast group had responded to the questionnaire in same classroom on a built environment.

Contrarily, experience group answered the test immersed in nature after an excursion in Isla Del Tiburon, where they took a 2-mile walk along the island watching local desertic flora and fauna and being surrounded by mountains and the sea. At boarding area, sitting on sand and under palm trees participants answered to the printed questionnaire by hand in approximately 15 min.

### Data Analysis

Firstly, instrument reliability was tested by Cronbach’s Alpha (α). Then, collected data was analyzed using statistical package SPSS version 21.0, through an exploratory factorial analysis, descriptive statistics, independent samples *T*-test t and correlates between variables were computed.

## Results

A reliability test was executed indicating good consistency between instrument application in both groups as well as periods (Table 1). Data analysis was performed, and as H1 predicted, no differences were found in pre-excursion section in between groups referring that indeed, both groups have similar feelings toward nature and were feeling satisfied at the moment. As H2 established, some constructs of NC and wellbeing registered an increasement regarding the experience group in the second phase due to higher results on INS, CNS, NR, and LCN for NC variables. Also, PW and PANAS related to the wellbeing construct, showing differences with the contrast group at the same stage.

**TABLE 1:** Cronbach’s Alpha reliability test.

	Pre-excursion		Post-excursion	
Scale	Contrast	Experience	Contrast	Experience
Positive affects schedule (PANAS)	0.851	0.866	0.838	0.916
Negative affects schedule (PANAS)	0.894	0.823	0.830	0.862
Nature connectedness scale	0.933	0.781	0.929	0.875
Nature relatedness scale	0.838	0.783	0.847	0.850
Love and care for nature scale	0.947	0.956	0.968	0.953
Satisfaction with life scale	0.0873	0.845	0.919	0.866

INS frequencies (see Table 2), shows how related the person feels to nature at the moment at the moment they answered questionnaire, noticing higher frequencies in item 6 regarding the experience group on the post-excursion phase by difference to contrast group diversifying answers.

**TABLE 2:** Inclusion of nature in self item frequencies.

		1	2	3	4	5	6	7
		Self-nature	Self-nature	Self-nature	Self-nature	Self-nature	Self-nature	Self-nature
Phase	Group	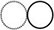	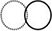	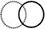	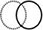	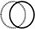	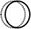	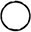
Pre-excursion	Contrast	0	1	6	8	9	6	2
	Experience	0	3	2	12	5	6	1
Post-excursion	Contrast	0	3	5	6	8	7	3
	Experience	0	0	1	4	8	13	3

Descriptive statistics were computed for each group by obtaining M and Standard deviation from both groups and phases as displayed (see Table 3). As dividing PANAS in two sections, Positive affects from PANAS indicate experience group registered responses such as having more positive emotional affects when answering the questionnaire surrounded by nature. As for the post-excursion phase results also showed reduced negative affects, meaning they felt less aggressive, anxious, and anguished than our contrast group in this phase. In SWL, the experience group seemed to be more satisfied with their lives, their own choices and circle of friends than the contrast group. NCS showed that the experience group had a closer experience with nature, feeling part of the natural world and of the life cycle. NR indicated that the experience group displayed a greater preference about being surrounded by nature and feeling more comfortable in this environment than the other group. LCN showed that contrast group scored *M* = 5.40 and the experience group *M* = 5.91.

**TABLE 3:** *T*-test results.

Scale	Phase	Group	*M*	*t*	*d.f.*	*p*	*r*
Positive affects (PANAS)	Pre-excursion	Contrast	3.44	–0.83	59	0.67	
		Experience	3.58		59		
	Post-excursion	Contrast	3.56	–3.47	59	0.00	0.10
		Experience	4.00		59		
	Pre-excursion	Contrast	4.45	0.15	59	0.58	
Negative affects (PANAS)		Experience	4.43		59		
	Post-excursion	Contrast	4.45	4.02	59	0.00	0.001
		Experience	3.76		59		
	Pre-excursion	Contrast	5.40	–1.79	59	0.74	
Satisfaction with life							
		Experience	5.81		59		
	Post-excursion	Contrast	5.41	–1.70	59	0.09	0.22
		Experience	5.84		59		
	Pre-excursion	Contrast	5.25	–0.57	59	0.25	
Nature connectedness							
		Experience	5.39		59		
	Post-excursion	Contrast	5.37	–1.21	59	0.22	0.07
		Experience	5.68		59		
Nature relatedness	Pre-excursion	Contrast	4.86	–1.28	59	0.59	
		Experience	5.18		59		
	Post-excursion	Contrast	4.99	–2.07	59	0.04	0.03
		Experience	5.48		59		
	Pre-excursion	Contrast	5.40	–0.17	59	0.96	
Love and care for nature							
		Experience	5.45		59		
	Post-excursion	Contrast	5.40	–2.08	59	0.04	0.02
		Experience	5.91		59		
	Pre- excursion	Contrast	5.38	1.98	59	0.83	
Psychological wellbeing		Experience	4.97		59		
	Post-Excursion	Contrast	5.27	0.29	59	0.77	0.24
		Experience	5.21		59		

This result goes to show that even if the contrast group had a moderately high M score, the experience group had a higher level of feeling passionate for the natural world, more spiritually close to Earth, and caring more the planet when being surrounded by nature. PW in contrast group obtained *M* = 5.38 and the experience group *M* = 5.21 demonstrating contrast group has a higher level of satisfaction about their achievements and objectives as people. Also, to compare means between samples, an independent sample *T*-test was made reporting significant comparison between contrast and experience group means on PANAS, LCN and NR each on the second phase (see Table 3).

Correlates indicate an outstanding interrelation (see Table 4) of LCN and NR as well as the correlation of LCS with NCS and NR with NCS. Also, to be highlighted are the strong correlates between NR and SWL, SWL and NCS, and SWL and LCN when testing study variables.

**TABLE 4:** Variable correlates.

Scale	PANAS	SWLS	NCS	NRS	LCN	PWS
Positive and negative affects schedule						
Satisfaction with life scale	0.147					
Nature connectedness scale	0.085	0.366**				
Nature relatedness scale	0.199	0.404**	0.674**			
Love and care for nature scale	0.241	0.319*	0.703**	0.797**		
Psychological wellbeing scale	0.239	0.223	0.149	0.181	0.104	

*p < 05, **p < 0.001. PANAS, Positive and Negative Affects Scale; NC, Nature Connectedness; NR, Nature Relatedness; LCN, Love and Carefor Nature; SWL, Satisfaction with Life; PW, Psychological Wellbeing.

## Discussion

The findings in this study imply that being surrounded by natural environments actually contributes to improving individual levels of NC, emotions, and wellbeing. In particular, Isla Del Tiburon presents peculiar conditions that provide positive elements to people as participants in this study reported significant results in some scales. Data collection settings between the two groups differed markedly in order to encompass the impact that the experience group had on its persona and nature concerns, as opposed to the contrast group which responded to the questionnaire in a regular indoor classroom environment.

This study offers a broader panorama of Environmental Psychology and Ecopsychology, which ensures that people benefit from being in nature. These findings also contribute to the fact that arid regions such as the Sonoran desert, despite the hot weather and exotic vegetation, actually provide a large list of assets to human feelings and insights.

The experience group got higher scores in PANAS regarding an improved SW compared to contrast group in the post-excursion phase. Participants who went to an excursion outdoors scored higher on LCN and NR, as well as higher feelings of INS. Both variables registered positive results, which means that excursioning in Isla Del Tiburon heightened the feeling of loving and caring for Nature as explained by Perkins (2010), as well as their own relationship with nature, feeling spiritually connected to the Earth and having a notion about its care (Nisbet et al., 2009). But this we can remark Greenway (1995), who refers after a study that strolling or exercising in a natural environment contributes to the improvement in individual’s functioning and attention; reduces anxiety, depression, and stress; and generates higher levels of happiness than when performing activities in urban or built places (Ulrich, 1984; Berman et al., 2008; Nisbet and Zelenski, 2011; Izenstark et al., 2021).

Data also showed that PANAS, after being separated into positive and negative affects, indicates significant differences in participants who performed an activity in a natural environment than those who did not; meaning that they felt more at ease, happy and comfortable in nature. Choe et al. (2020) in the United Kingdom demonstrated in a study (*n* = 122) that people reported considerable rising levels of wellbeing after being exposed to a natural environment as part of a relaxation program. Findings of Watson et al. (1988) despite of mentioning the concerns and limitations that could have obstructed individuals from feeling openly happy and revitalized indicate that results were presented being related to negative aspects that diminished in the experience group like reducing emotions (e.g., anguish, guilt, aggressiveness, and irritability). In Guadeloupe, Robin et al. (2021) measured positive and negative aspects after being in a natural zone aiming to discover the emotional effects of being surrounded by a tropical environment, also an innovative setting where nature connectedness was assessed, presenting a climate with humid weather characteristics. Although they found that participants indicated negative feelings (e.g., fatigue, discomfort) after being surrounded by this climate, generating unpleasant emotions on people. According to the present study, some conditions might appear to have complicated the excursionists’ mood and development during the experience on the Island. These are related to people who were not used to hiking or taking long walks and also low tolerance to high sun exposure, being tired, hungry or some other conditions.

Limitations on this study refer on at least three conditions that relate to sample size. Firstly, due to travel cost, visitor expenses must be provided limiting to cover a large group of visitors. Also, duration of the excursion (whole journey) may also skew the number of participants, therefore making it complicated for people to join such experience as they have busy days or other scheduled activities. Lastly, limited number of visitors are allowed by the reserve residents. Thus, the Island is not open to public except if visitors join a recognized tour agency or a permitted group with a prescheduled visit. This suggested the research team to consider a very selected sample.

Additional research is suggested to profound on the impact people may have from being in contact with nature. Further inquiries should focus on each person enhance, reflections, deep emotions or cognitive affections while being outdoors. Also seizing from various activities, different settings should be considerate in order to promote better and steady contact with nature as well as major findings related to the self and nature bond.

After data analysis, benefits were found on the experience group concerning the way nature is perceived and the relationship they develop creating a tighter human-nature bond and feeling part of natural world. These two aspects regain value nowadays after experiencing home confinement caused by the COVID-19 pandemic, where people are forced to live not only in total social isolation but also deprived of nature and by limiting visits to green, natural, or outdoor spaces therefore feeling the effects of these restrictions in many ways. Also, it is important to keep exploring and analyzing nature benefits and promoting people to consciously enjoy the environment and recover from the large pandemic that affected the entire world.

## Data Availability Statement

The raw data supporting the conclusions of this article will be made available by the authors, without undue reservation.

## Ethics Statement

The studies involving human participants were reviewed and approved by Comité de Ética en Investigación de la Universidad de Sonora. Written informed consent to participate in this study was provided by the participants’ legal guardian/next of kin. The patients/participants provided their written informed consent to participate in this study. Written informed consent was obtained from the individual(s) for the publication of any identifiable images or data included in this article.

## Author Contributions

GG-T contributed with the conceptualization, and design of this study, acquisition of data, ran formal analysis, and organized databases. CT-F contributed by supervising this study, its methodological tasks, and data interpretation. BF-S and LP made substantial contributions by editing and revising the manuscript critically for important intellectual content. GG-T and DB-M provided the writing of the original draft. All authors contributed to manuscript revision and read and approved the submitted version.

## Conflict of Interest

The authors declare that the research was conducted in the absence of any commercial or financial relationships that could be construed as a potential conflict of interest.

## Publisher’s Note

All claims expressed in this article are solely those of the authors and do not necessarily represent those of their affiliated organizations, or those of the publisher, the editors and the reviewers. Any product that may be evaluated in this article, or claim that may be made by its manufacturer, is not guaranteed or endorsed by the publisher.

## References

[B1] Acosta G. (2002). Seris de Sonora. Proyecto Perfiles Indígenas de México, Documento de Trabajo.

[B2] Ameli R. Skeath P. Abraham P. A. Panahi S. Kazman J. B. Foote F. (2021). A nature-based health intervention at a military healthcarecenter: a randomized, controlled, cross-over study. *Peer J.* 9:e10519. 10.7717/peerj.10519 33505785 PMC7789867

[B3] Aragonés J. I. Amérigo M. (2000). Psicología Ambiental. Madrid: Ediciones Pirámide.

[B4] Balundė A. Perlaviciute G. Steg L. (2019). The relationship between people’s environmental considerations and pro-environmental behavior in Lithuania. *Front. Psychol.* 10:2319. 10.3389/fpsyg.2019.02319 31681111 PMC6803424

[B5] Barrable A. Booth D. (2020). Increasing nature connection in children: A mini review of interventions. *Front. Psychol.* 11:492. 10.3389/fpsyg.2020.00492 32265797 PMC7096575

[B6] Berman M. G. Jonides J. Kaplan S. (2008). The cognitive benefits of interacting with nature. *Psychol. Sci.* 19 1207–1212. 10.1111/j.1467-9280.2008.02225.x 19121124

[B7] Biedenweg K. Scott R. P. Scott T. A. (2017). How does engaging with nature relate to life satisfaction? Demonstrating the link between environment-specific social experiences and life satisfaction. *J. Environ. Psychol.* 50 112–124.

[B8] Bowler D. E. Buyung-Ali L. M. Knight T. M. Pullin A. S. (2010). A systematic review of evidence for the added benefits to health of exposure to natural environments. *BMC Public Health* 10:456–466. 10.1186/1471-2458-10-456 20684754 PMC2924288

[B9] Brügger A. Kaiser F. G. Roczen N. (2011). One for all? : connectedness to nature, inclusion of nature, environmental identity, and implicit association with nature *Eur. Psychol.* 16 324–333

[B10] Capaldi C. A. Dopko R. L. Zelenski J. M. (2014). The relationship between nature connectedness and happiness: A meta-analysis. *Front. Psychol.* 5:976. 10.3389/fpsyg.2014.00976 25249992 PMC4157607

[B11] Choe E. Y. Jorgensen A. Sheffield D. (2020). Does a natural environment enhance the effectiveness of Mindfulness-Based Stress Reduction (MBSR)? Examining the mental health and wellbeing, and nature connectedness benefits. *Land. Urban Plann.* 202:103886.

[B12] Collado S. Corraliza J. A. (2016). Conciencia Ecológica y Bienestar en la Infancia. Efectos de la Relación con la Naturaleza. Madrid: CCS, 77–122.

[B13] Crocker P. R. (1997). A confirmatory factor analysis of the positive affect negative affect schedule (PANAS) with a youth sport sample. *J. Sport Exerc. Psychol.* 19 91–97 10.1123/jsep.19.1.91

[B14] Diener E. (1994). Assessing subjective well-being: Progress and opportunities. *Soc. Indicat. Res.* 31 103–157. 10.1007/BF01207052

[B15] Diener E. D. Emmons R. A. Larsen R. J. Griffin S. (1985). The satisfaction with life scale. *J. Personal. Assess.* 49 71–75. 10.1207/s15327752jpa4901_13 16367493

[B16] Duerden M. D. Witt P. A. (2010). The impact of direct and indirect experiences on the development of environmental knowledge, attitudes, and behavior. *J. Environ. Psychol.* 30 379–392. 10.1016/j.jenvp.2010.03.007

[B17] Fong K. C. Hart J. E. James P. (2018). A review of epidemiologic studies on greenness and health: Updated literature through 2017. *Curr. Environ. Health Rep.* 5 77–87. 10.1007/s40572-018-0179-y 29392643 PMC5878143

[B18] Greenway R. (1995). “The wilderness effect and ecopsychology”. *Ecopsychology: Restoring the earth, healing the mind.* Kanner A. D. Gomes M. E. Roszak T. (eds) (San Francisco: Sierra Club Books)

[B19] Hansen M. M. Jones R. Tocchini K. (2017). Shinrin-yoku (forest bathing) and nature therapy: A state-of-the-art review. *Int. J. Environ. Res. Public Health* 14:851. 10.3390/ijerph14080851 28788101 PMC5580555

[B20] Hinds J. Sparks P. (2008). Engaging with the natural environment: The role of affective connection and identity. *J. Environ. Psychol.* 28 109–120. 10.1016/j.jenvp.2007.11.001

[B21] Howell A. J. Dopko R. L. Passmore H.-A. Buro K. (2011). Nature connectedness: Associations with well-being and mindfulness. *Personal. Indiv. Diff.* 51 166–171. 10.1016/j.paid.2011.03.037

[B22] Howell A. J. Passmore H. (2013). “The nature of happiness: Nature affiliation and mental well being,” in *Mental Well-Being: international Contributions to the Study of Positive Mental Health*, ed. Keyes C. L. M. (New York: Springer), 231–257. 10.1007/978-94-007-5195-8_11

[B23] Izenstark D. Ravindran N. Rodriguez S. Devine N. (2021). The affective and conversational benefits of a walk in nature among mother–daughter dyads. *Appl. Psychol.* 13 299–316 10.1111/aphw.12250 33755327

[B24] Kaplan R. (1993). The role of nature in the context of the workplace. *Land. Urban plann.* 26 193–201. 10.1016/0169-2046(93)90016-7

[B25] Kaplan R. Kaplan S. (1989). *The experience of nature: A psychological perspective.* Cambridge:Cambridge University Press.

[B26] Kaplan S. Talbot J. F. (1983). *Psychological Benefits of a Wilderness Experience. In Behavior and the Natural Environment.* Boston, MA: Springer, 163–203. 10.1007/978-1-4613-3539-9_6

[B27] Kasap E. Z. Aðzıtemiz F. Ünal G. (2021). Cognitive, mental and social benefits of interacting with nature: A systematic review. *J. Happiness Health* 1 16–27.

[B28] Kotera Y. Richardson M. Sheffield D. (2020). Effects of shinrin-yoku (forest bathing) and nature therapy on mental health: a systematic review and meta-analysis. *Int. J. Ment. Health Addict.* 20, 337–361. 10.1007/s11469-020-00363-4

[B29] Lim P. Y. Dillon D. Chew P. K. (2020). A guide to nature immersion: psychological and physiological benefits. *Int. J. Environ. Res. Public Health* 17:5989. 10.3390/ijerph17165989 32824731 PMC7459647

[B30] Loureiro A. Veloso T. J. (2014). Outdoor exercise, well-being and connectedness to 443 nature. *Psico* 45 299–304. 10.15448/1980-8623.2014.3.19180

[B31] Martin C. Czellar S. (2016). The extended inclusion of nature in self scale. *J. Environ. Psychol.* 47 181–194. 10.1016/j.jenvp.2016.05.006

[B32] Mayer F. S. Frantz C. M. (2004). The connectedness to nature scale: A measure of individuals’ feeling in community with nature. *J. Environ. Psychol.* 24 503–515. 10.1371/journal.pone.0249890 33878132 PMC8057610

[B33] McMahan E. A. Estes D. (2015). The effect of contact with natural environments on positive and negative affect: A meta-analysis. *J. Posit. Psychol.* 10 507–519. 10.1080/17439760.2014.994224

[B34] Millar M. G. Millar K. U. (1996). The effects of direct and indirect experience on affective and cognitive responses and the attitude–behavior relation. *J. Exper. Soc. Psychol.* 32 561–579. 10.1006/jesp.1996.0025 8979934

[B35] Moral R. J. (2011). La escala de afecto positivo y negativo (PANAS) en parejas casadas mexicanas. *Ciencia Ergo Sum* 18 117–125.

[B36] Morita E. Fukuda S. Nagano J. Hamajima N. Yamamoto H. Iwai Y. (2007). Psychological effects of forest environments on healthy adults: Shinrinyoku (forest-air bathing, walking) as a possible method of stress reduction. *Public Health* 121 54–63. 10.1016/j.puhe.2006.05.024 17055544

[B37] Müller M. M. Kals E. Pansa R. (2009). Adolescents’ emotional affinity toward nature: A cross-societal study. *J. Dev. Processes* 4 59–69.

[B38] National Research Council (2013). *Subjective Well-Being: Measuring Happiness, Suffering, and Other Dimensions of Experience*. Washington, DC: The National Academies Press. 10.17226/18548 24432436

[B39] Nisbet E. K. Zelenski J. M. (2011). Underestimating nearby nature: Affective forecasting errors obscure the happy path to sustainability. *Psychol. Sci.* 22 1101–1106. 10.1177/0956797611418527 21828351

[B40] Nisbet E. K. Zelenski J. M. Murphy S. A. (2009). The nature relatedness scale: Linking individuals’ connection with nature to environmental concern and behavior. *Environ. Behav.* 41 715–740. 10.1177/0013916508318748

[B41] Olivos P. Aragonés J. I. Amérigo M. (2011). The connectedness to nature scale and its relationship with environmental beliefs and identity. *Int. J. Hispanic Psychol.* 4 5–19.

[B42] Olivos P. Clayton S. (2017). “Self, nature and well-being: Sense of connectedness and environmental identity for quality of life,” in *Handbook of environmental psychology and quality of life research*, Pol E. Fleury-Bahi G. Navarro O. (Cham: Springer), 107–126. 10.1007/978-3-319-31416-7_6

[B43] Olivos P. Aragonés J. I. (2014). Medio ambiente, self y conectividad con la naturaleza. *Revista Mexicana Psicología* 31 71–77.

[B44] Pasca L. Aragonés J. I. Coello M. T. (2017). An analysis of the connectedness to nature scale based on item response theory. *Front. Psychol.* 8:1330. 10.3389/fpsyg.2017.01330 28824509 PMC5543030

[B45] Perkins H. E. (2010). Measuring love and care for nature. *J. Environ. Psychol.* 30 455–463. 10.1016/j.jenvp.2010.05.004

[B46] Pirchio S. Passiatore Y. Panno A. Cipparone M. Carrus G. (2021). The Effects of Contact With Nature During Outdoor Environmental Education on Students’ Wellbeing, Connectedness to Nature and Pro-sociality. *Front. Psychol.* 12:648458. 10.3389/fpsyg.2021.648458 34017288 PMC8129515

[B47] Pritchard A. Richardson M. Sheffield D. McEwan K. (2020). The relationship between nature connectedness and eudaimonic well-being: A meta-analysis. *J. Happiness Stud.* 21 1145–1167. 10.1371/journal.pone.0203000 30208073 PMC6135392

[B48] Reese G. Kohler E. Menzel C. (2021). Restore or get restored: The effect of control on stress reduction and restoration in virtual nature settings. *Sustainability* 13:1995. 10.3390/su13041995

[B49] Restall B. Conrad E. (2015). A literature review of connectedness to nature and its potential for environmental management. *J. Environ. Manage.* 159 264–278. 10.1016/j.jenvman.2015.05.022 26087657

[B50] Robin N. Sinnapah S. Hue O. Coudevylle G. R. (2021). Tropical climate influences affects, sensation of fatigue and environmental perceptions (El clima tropical influye sobre los afectos, la sensación de fatiga y las percepciones del medio ambiente). *PsyEcology* 12 1–25. 10.1080/21711976.2021.1954439

[B51] Robles R. Páez F. (2003). Estudio sobre la traducción al español y las propiedades psicométricas de las escalas de afecto positivo y negativo (PANAS). *Salud mental* 26 69–75.

[B52] Rojas O. R. Puebla F. Figueroa E. M. Nakazawa Y. J. Ríos C. A. Navarro A. G. (2002). Avifauna de Isla Tiburón, Sonora, México. Anales del Instituto de Biología. *Serie Zoología* 73 73–89.

[B53] Roy S. Byrne J. Pickering C. (2012). A systematic quantitative review of urban tree benefits, costs, and assessment methods across cities in different climatic zones. *Urban Forestry Urban Green.* 11 351–363. 10.1016/j.ufug.2012.06.006

[B54] Russell R. Guerry A. D. Balvanera P. Gould R. K. Basurto X. Chan K. M. (2013). Humans and nature: how knowing and experiencing nature affect well-being. *Ann. Rev. Environ. Resour.* 38‘ 473–502. 10.1146/annurev-environ-012312-110838

[B55] Ryan R. Deci E. (2001). On happiness and human potentials: A review of research on hedonic and eudaimonic well-being. *Annu. Rev. Psychol.* 52 141–166. 10.1146/annurev.psych.52.1.141 11148302

[B56] Ryan R. M. Weinstein N. Bernstein J. Brown K. W. Mistretta L. Gagne M. (2010). Vitalizing effects of being outdoors and in nature. *J. Environ. Psychol.* 30 159–168 10.1016/j.jenvp.2009.10.009

[B57] Ryff C. D. (1989). Happiness is everything, or is it? Explorations on the meaning of psychological well-being. *J. Personal. Soc. Psychol.* 57 1069–1081. 10.1037/0022-3514.57.6.1069

[B58] Schultz P. W. (2001). The structure of environmental concern: Concern for self, other people, anthe biosphere. *J. Environ. Psychol.* 21 327–339. 10.1006/jevp.2001.0227

[B59] Schultz P. W. Shriver C. Tabanico J. J. Khazian A. M. (2004). Implicit connections with nature. *J. Environ. Psychol.* 24 31–42. 10.1371/journal.pone.0127247 25985075 PMC4436134

[B60] Seymour V. (2016). The human–nature relationship and its impact on health: A critical review. *Front. Public Health* 4:260. 10.3389/fpubh.2016.00260 27917378 PMC5114301

[B61] Simmons I. G. (1993). *Interpreting Nature.* London: Cultural Constructions of the Environment.

[B62] Song C. Ikei H. Kobayashi M. Miura T. Li Q. Kagawa T. (2017). Effects of viewing forest landscape on middle-aged hypertensive men. *Urban Forestry Urban 510 Green.* 21 247–252. 10.1016/j.ufug.2016.12.010

[B63] Tam K. P. (2013). Dispositional empathy with nature. *J. Environm. Psychol.* 35 92–104. 10.1016/j.jenvp.2013.05.004

[B64] Tamayo M. (1980). *Metodología formal de la investigación científica.* Mexico: Editorial Limusa.

[B65] Tauber P. G. (2012). *An Exploration of the Relationships Among Connectedness to Nature, Quality of Life, and Mental Health.* Logan, UT: Utah State University.

[B66] Terracciano A. McCrae R. R. Hagemann D. Costa P. T. Jr. (2003). Individual difference variables, affective differentiation, and the structures of affect. *J. Personal.* 71 669–704. 10.1111/1467-6494.7105001 12932207 PMC2580756

[B67] Ulrich R. S. (1984). View through a window may influence recoverfrom surgery. *Science* 224 420–421. 10.1126/science.6143402 6143402

[B68] Ward J. S. Duncan J. S. Jarden A. Stewart T. (2016). The impact of children’s exposure to greenspace on physical activity, cognitive development, emotional wellbeing, and ability to appraise risk. *Health Place* 40 44–50. 10.1016/j.healthplace.2016.04.015 27179137

[B69] Waterman A. S. (2008). Reconsidering happiness: A eudaimonist’s perspective. *J. Positive Psychol.* 3 234–252. 10.1080/17439760802303002

[B70] Watson D. Clark L. A. Tellegen A. (1988). Development and validation of brief measures of positive and negative affect: the PANAS scales. *J. personal. soc. psychol.* 54:1063. 10.1037/0022-3514.54.6.1063 3397865

[B71] Zevon M. A. Tellegen A. (1982). The structure of mood change: An idiographic/nomothetic analysis. *J. Personal. Soc. Psychol.* 43:111. 10.1037/0022-3514.43.1.111

