# Supplementary material for: Impact of Contact With Nature on the Wellbeing and Nature Connectedness Indicators After a Desertic Outdoor Experience on Isla Del Tiburon
Source: Front Psychol. 2022 Jun 3;13:864836. doi: 10.3389/fpsyg.2022.864836 (PMC9204234; doi:10.3389/fpsyg.2022.864836)
Supplement: Supplementary file 1 [file Data_Sheet_1.PDF]

Supplementary Material

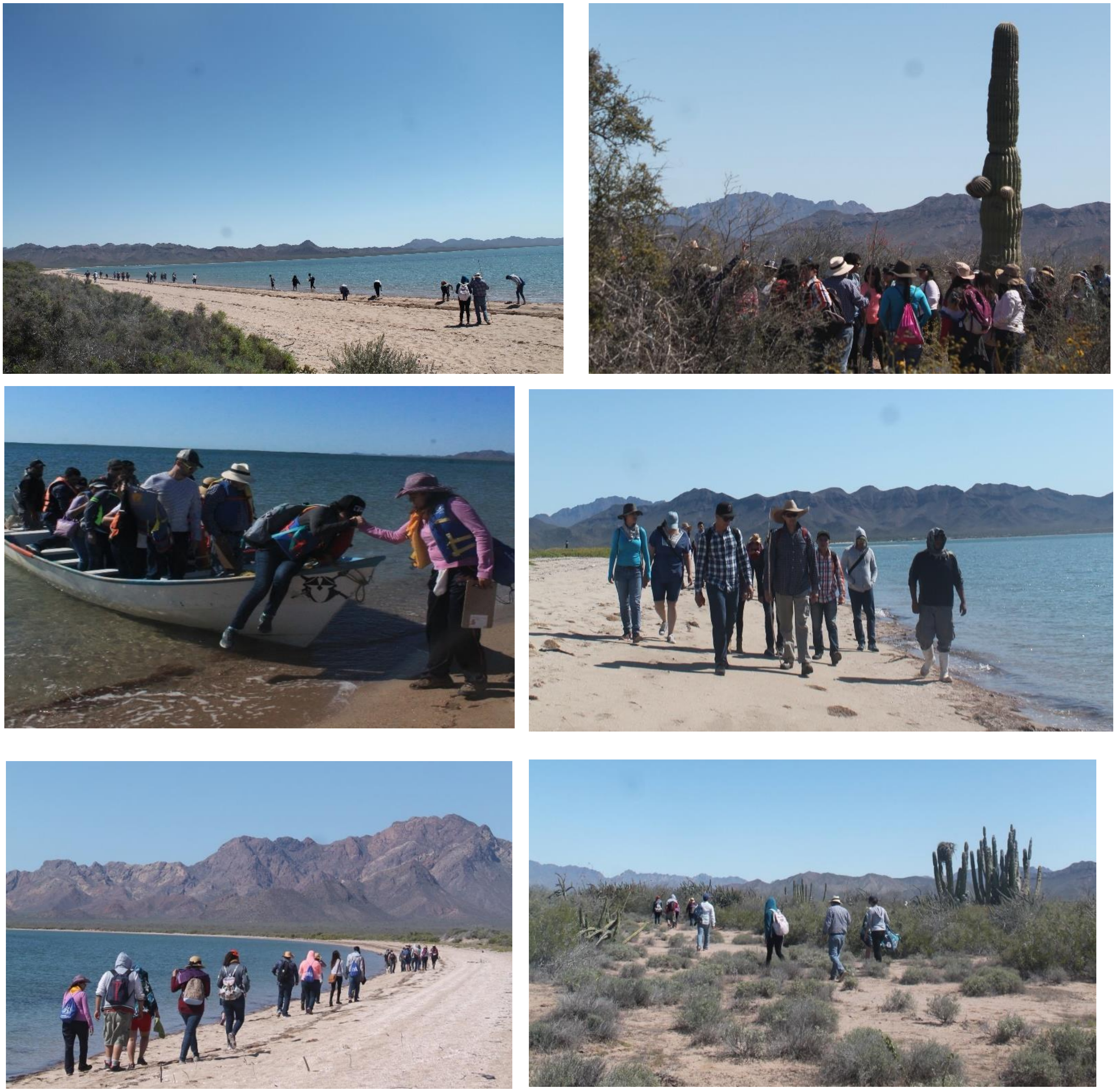

Pictures taken during the outdoor experience on Isla Del Tiburon

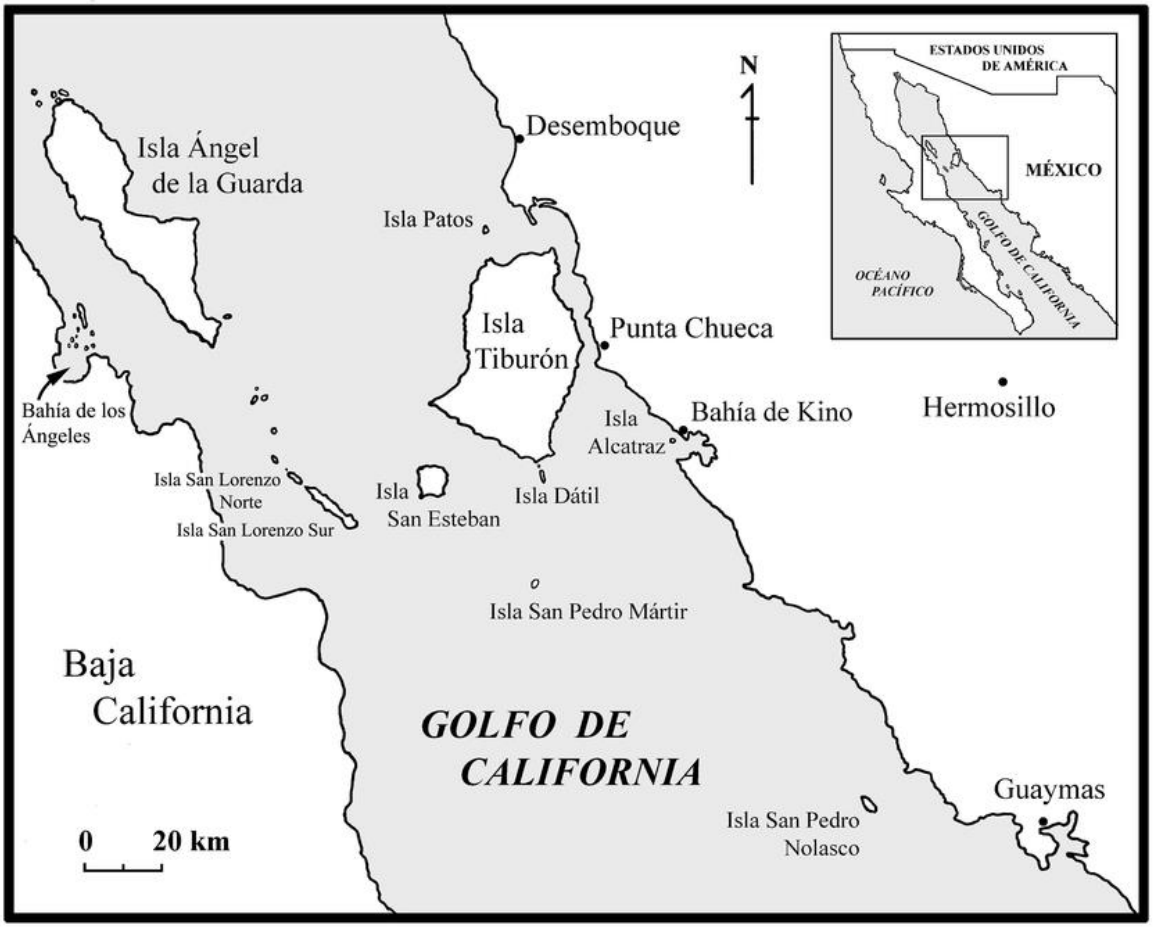

Supplementary figure 1. Isla del Tiburon Location. Map by Cathy Moser Marlett.
